# Robust, Fire-Retardant, and Water-Resistant Wood/Polyimide Composite Aerogels with a Hierarchical Pore Structure for Thermal Insulation

**DOI:** 10.3390/gels9060467

**Published:** 2023-06-06

**Authors:** Lu Zhao, Junyong Chen, Defang Pan, Yan Hou

**Affiliations:** School of Chemistry and Chemical Engineering, Qilu Normal University, Jinan 250200, China

**Keywords:** wood, polyimide, hydrophobicity, thermal insulation, fire-retardant

## Abstract

The use of energy-saving materials is an effective strategy for decreasing energy consumption and carbon emission. Wood is a type of biomass material with a natural hierarchical structure, which results in its high thermal insulation. It has been widely used in construction. However, developing wood-based materials without flammability and dimensional instability is still a challenge. Herein, we developed a wood/polyimide composite aerogel with a well-preserved hierarchical pore structure and dense hydrogen bonds inside, resulting in its excellent chemical compatibility and strong interfacial interactions between its two components. This novel wood-based composite was fabricated by removing most hemicellulose and lignin from natural wood, followed by the fast impregnation using an ‘in situ gel’ process. The introduction of polyimide into delignified wood substantially improved its mechanical properties, with the compression resistance being improved by over five times. Notably, the thermal conductivity coefficient of the developed composite was approximately half that of natural wood. Furthermore, the composite exhibited excellent fire-retardancy, hydrophobicity, thermal insulation, and mechanical properties. This study provides a novel method for wood modification, which not only aids interfacial compatibility between wood and polyimide but also retains the properties of the two components. The developed composite can effectively reduce energy consumption, making it promising for practical and complex thermal insulation applications.

## 1. Introduction

Reducing heat loss is crucial for saving energy [1]. Currently, commercial furniture materials, such as wool, expanded polystyrene (EPS), and polyurethane (PU) foams, are widely used as thermal insulators for reducing energy consumption. Moreover, wood has attracted considerable attention as a renewable and sustainable thermal insulator with excellent mechanical strength [2,3]. However, it adsorbs water, making it easily biodegradable. In addition, the flammability of wood poses considerable safety risks. These issues have hindered the development of wood products [4].

Wood primarily comprises cellulose fibril aggregates, hemicellulose, and lignin, which intertwine tightly to form an open lumen that can transport nutrients and water [5]. The packed nanofibers constitute the wood cell walls, and each fibril with high aspect ratios composes several tens of glucan chains, which formed intermolecular hydrogen bonds and interacted through van der Waals forces [6]. The microstructure and hierarchal alignment of wood are well maintained.

However, flammability is a well-known disadvantage of wood-based materials. Flame retardants, such as brominated flame retardants and phosphorus-containing compounds, are commonly used to modify wood to enhance its flame retardancy [7]. The limiting oxygen index (LOI) and thermal degradation kinetics are commonly employed to evaluate the performance of flame retardants. The LOI is a measure of the difficulty of burning a substance, where the higher the LOI is, the more difficult a substance it is to burn. Thermal degradation kinetics can be used to analyze the high-temperature resistance of flame retardants. In addition, various silica modifiers have been explored for wood treatment [8,9,10,11]. For example, tetraethoxysilane, 2-heptadecafluorooctylethyltrimet-hoxysilane, methyltrimethoxysilane, and polydimethylsiloxane have been used as precursors by sol-gel method to prepare inorganic wood-based composites [12]. Silica aerogels have extremely low thermal conductivities of ~0.02 W/(m·K), making them excellent inorganic thermal insulators [13,14]. However, silica aerogels are brittle and have poor mechanical properties, which inhibited their widespread applications on a larger scale [15]. In contrast, many organic compounds exhibit better mechanical properties than silica modifiers. They are used to prepare cellulose-based materials with good flame retardancy. For example, Guo et al. [16] prepared a hierarchical CNF-HAP composite foam, where HAP provided thermal degradation resistance. Han et al. [17] fabricated a CNF/polydopamine/Fe^3+^ aerogel, in which the functionalization of CNF by polydopamine and crosslinking by Fe^3+^ considerably improved the thermal stability of the material. Additionally, polyimide (PI) aerogels have attracted considerable attention owing to their excellent mechanical properties and high-temperature stability, and have also been shown to effectively contribute to flame-retardancy [18,19]. Chen et al. [20] used microscale combustion calorimetry to investigate the combustion behavior of a wood/PI composite and identified the critical parameters for combustion performance, including the heat release rate (HRR) and peak HRR (PHRR). Incorporating PI into the composite results in the formation of a char layer on the residue after cyclic combustion, preventing heat transfer between the flame and composite. Ren et al. [21] conducted a combustion test and compared the peak smoke production rate (PSPR), PHRR, and total HRR (THRR), proving that PI incorporation can prevent oxygen from penetrating into wood during combustion and promote the formation of surface carbon, resulting into the composite exhibiting combustion delay and smoke suppression.

However, it is difficult for PI with high molecular weights to directly permeate into wood. Thus, a vacuum-assisted impregnation strategy has been widely employed in polymer systems with poor mobility, high viscosity, and large molecular weight [22,23]. Zhang et al. adopted a vacuum-pressure impregnation process to infiltrate conductive particles into wood channels [24]. Zhu et al. infiltrated epoxy resin polymers into wood microstructures under vacuum cycling [25]. However, vacuum pressure can deform the wood skeleton in real operations. Thus, further improvement is needed in the fabrication process to preserve the hierarchal arrangement of natural wood (NW), which induces low thermal conductivity along the cellulose alignment direction resulting from the strong covalent bonds inside wood cell walls and the tight combination of three components (cellulose, hemicellulose, and lignin). Recently, chemical treatments have been used to process various wood-based composites [26]. For example, Chen et al. [27] extracted cellulose from balsa wood by removing lignin and most of the hemicellulose (via treatment with NaOH and NaHSO_3_ at 90 °C for 12 h). Guan et al. [28] fabricated wood sponges using NaClO_2_ and NaOH solutions to remove the lignin and hemicellulose fractions from the wood cell wall.

**Figure 1:**
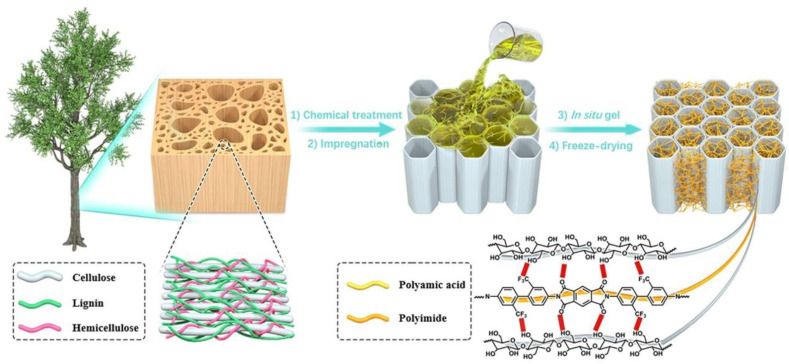
Schematic of the fabrication of wood/polyimide aerogel.

Inspired by these ‘top-down’ processes, herein, we designed a strategy for modifying wood via the in situ polymerization of PI in delignified wood (DW). An ‘in situ gel’ approach was employed to ensure that the formed PI chains are cross-linked to intertangled three-dimensional (3D) fibrillar networks in layered porous DW. Taking balsa wood as an example (Figure 1), lignin and hemicellulose were partly removed from NW to decrease the density, resulting in a more open lumen, which facilitated subsequent impregnation. With the careful regulation of chemical treatment and the freeze-drying process, the main fiber components and pore structure were well-preserved [29]. Then, the DW sample was impregnated with a polyamic acid (PAA) solution until the white DW turned translucent, and then the DW/PI composite was subsequently obtained through the in situ polymerization of PAA in the presence of a catalyst (acetic anhydride and pyridine). Finally, the freeze-drying procedure was followed to obtain the DW/PI composite. For the obtained composite, the PI component acts as ‘lignin-like’ material, forming an interpenetrating fibrillar 3D network with cellulose molecules, thereby endowing DW/PI wood with outstanding mechanical properties (radial compressive strength is 1.25 MPa, when the elastic strain of materials ε = 60%) and lower thermal conductivity of 0.034 W/(m·K) in the radial direction, which was considerably lower than that of NW (0.066 W/(m·K)). The composite also exhibited excellent hydrophobicity (water contact angle of 125°), fire retardancy, and self-extinguishing properties. The lightweight, hydrophobic, robust, and fire-retardant wood/PI composite has broad application prospects in complex thermal insulation circumstances. Moreover, this novel procedure can be adapted to other complex systems.

## 2. Results and Discussion

### 2.1. Microstructure and Composition

**Figure 2:**
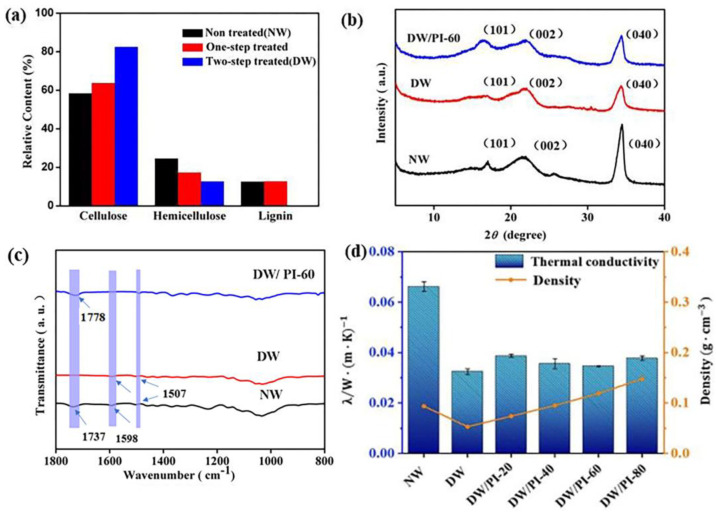
(**a**) Chemical composition of NW, one-step treated wood, DW; (**b**) XRD patterns of NW, DW, and DW/PI−60 composite: 2θ of 16.1°, 22.2°, and 34.8° correspond to (101), (002), and (040), respectively [21,25]; (**c**) FT−IR spectra of the NW, DW, and DW/PI-60 composite; (**d**) densities and thermal conductivity of the samples.

The fabrication process of DW/PI composite is illustrated in Figure 1. The obtained samples with different PI contents were labelled as DW/PI-x. Here, x refers to the concentration of polyimide precursor solution (x = 20, 40, 60, and 80 mg/cm^3^). Moreover, natural balsa wood was selected, which had a hierarchical and porous alignment structure for the transportation of water and nutrients to a whole plant, and an intertwined network of three components: cellulose, hemicellulose, and lignin (Figure S1a of Supplementary Materials). The two-step chemical treatment of NW reported in our previous study was employed herein [30]. After the treatment, lignin and hemicellulose were selectively removed, and their contents were reduced to 0% and 12.7%, respectively (Figure 2a). Then, the NaClO_2_ solution was added to remove the remaining lignin (Figure S2 of Supplementary Materials). During this procedure, NaClO_2_ (PH = 4.7 with acetic acid) released ClO_2_ when heated and reacted with lignin. Owing to lignin decomposition, light-absorbing aromatic rings were almost completely removed and the yellowish NW turned white. The decreased mechanical strength can be attributed to the lack of adhesion of lignin and hemicellulose. However, the aligned lumen of the resulting DW, which was mainly composed of cellulose fibril aggregates, was still well-preserved, which contributed to maintaining the strong mechanical property. Owing to the following ice crystal nucleation and growth, which contributes to causing the lumina cell to partially break, the thin cell collapsed into a layered wave structure after freeze drying (Figure S1b of Supplementary Materials). Figure 2b presents the X-ray diffraction (XRD) patterns of NW, DW, and DW/PI-60. DW/PI-60 exhibited prominent diffractions at 2θ=16.1°, 22.2°, and 34.8°, corresponding to (101), (002), and (040), respectively, indicating that the cellulose retained its crystalline structure [21,25]. Additionally, the exposed nanopores between isolated fiber on DW considerably reduced the transverse thermal conductivity, and the presence of layer-by-layer open channels subsequently ensured the complete impregnation of PAA.

PI aerogel was synthesized via the sol-gel process. After the precursors formed long chains and before closing the loop, the obtained PAA was impregnated into limited timber pipes, and then the chemical gel process was completed with the acetic anhydride and pyridine catalysts. The ‘in situ gel’ strategy enabled in situ polymerization of PI with wood. After the introduction of PI, the density of the wood/PI-40 was comparable to that of NW. The surface chemistry of the composite was determined via FT-IR (Fourier transform-infrared spectrometer) spectroscopy (Figure 2c). The absorption peak at 1507 cm^−^^1^ can be attributed to the vibration of the benzene groups in the lignin, and the peak at 1598 cm^−^^1^ can be attributed to the carbonyl aldehyde in the lignin [31]. The characteristic peak of xylan absorption was detected in 1737 cm^−^^1^ [32]. Owing to the partial removal of lignin and hemicellulose, the intensities of these peaks were decreased. The characteristic peak at 1778 cm^−1^ corresponded to the C=O group in PI [25,33], indicating that PI aerogel filled the lumen of DW. Moreover, as shown in XRD patterns of DW/PI-60, the intensity of the peak corresponding to cellulose (040) was low, which can be ascribed to the introduction of PI (Figure 2b). The polyimide molecules formed on the surface of wood can form hydrogen bonds with cellulose molecules [34], which affects the orderly arrangement of cellulose, and the diffractions of cellulose is weakened.

**Figure 3:**
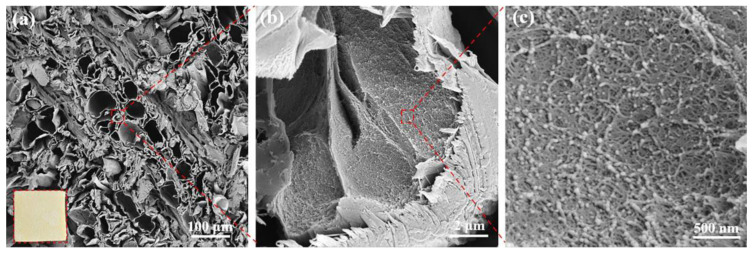
SEM images of DW/PI-60 aerogel with different magnifications; the scales are: (**a**) 100 μm, (**b**) 2 μm, and (**c**) 500 nm, respectively; the inset is an optical photograph of DW/PI-60 aerogel.

Wood/PI composite aerogels mainly exhibited a hierarchical pore structure, which resulted from the cross-linked PAA chains formed in the 3D nano-scale network and the intercellular micrometer-scale open channels. The PI aerogel molecular chains intertwined with the directionally aligned wood cellulose nanofiber to form an interpenetrated binary 3D network, enhancing chemical compatibility (Figure 3b,c). The well-maintained, aligned transport channels enabled more infiltration of PI (Figure 3a). The dense networks of PI and cellulose fiber formed strong hydrogen bond interactions.

### 2.2. Mechanical Properties

**Figure 4:**
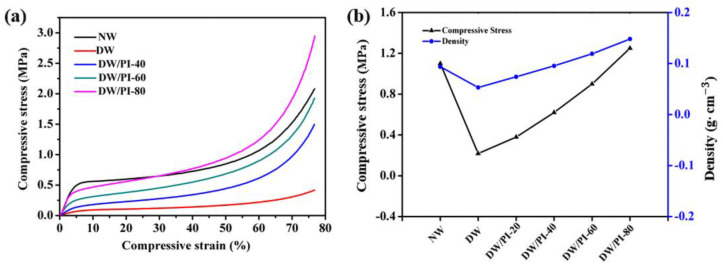
(**a**) Stress−strain curves of the samples; (**b**) elastic modulus and strength at 60% compression of the samples.

The stress–strain curves of the samples were obtained to evaluate the radical compressive strength of the materials (Figure 4a). The curves can be divided into three stages: linear elasticity, long strain yield, and densification stages. For NW, the strain (ε) of 10% represents the bending of cell walls, thus exhibiting a linear elastic stage. When ε is up to 40%, the curve changes slightly and the stress plateaus, which can be attributed to the compression of the aligned layered structure. When ε reached 60%, the radial compressive strength of NW was 1.1 Mpa. After the chemical delignination process, lignin was completely removed from the wood, the hemicellulose content decreased from 24.56% to 12.7% *(*Figure 2a), and the overall density of wood decreased significantly, and the compressive strength of wood decreased to 0.22 Mpa (ε=60%), only 20% of its original strength. As a result, the stress of DW decreases by 4.9 times compared with NW at 60% strain, indicating a significant decrease in mechanical strength. With the increased PI content, the radial compressive strength of the wood composite increased from 0.218 MPa of DW to 1.25 MPa of DW/PI-80, as shown in Figure 4b. Moreover, as shown in Table S2 of Supplementary Materials, the wood/PI-80 in this work has advantages of high strength and are much higher than other reported conventional thermal insulation materials, such as wood-waste foam [35], stone wool [36], and EPS foam [26]. Considering that cellulose is the dominant constituent of DW, the mechanism by which PI strengthens wood has two reasons: (1) at the molecular scale, a strong hydrogen bond exists between the PI chain and the hydroxyl groups in the cellulose molecular chains; and (2) the timber orientation structure is retained at the microscale, and the 3D networks of PI are filled into the cavity, resulting in a hierarchical structure, facilitating the provision of skeleton support.

### 2.3. Thermal Insulation

Figure 2d shows the comparison of the thermal conductivity of the samples. DW/PI-60, with a density of 0.119 g/cm^3^, shows a thermal conductivity of 0.034 W/(m·K), which is less than that of NW (0.066 W/(m·K)). In theory, the total thermal conductivity ($\lambda_{total}$) consists of convection, thermal conduction (where $\lambda_{g}$ represents gas conduction and $\lambda_{s}$ represents solid conduction), and thermal radiation ($\lambda_{r}$). Owing to the small pore size being well below that necessary for the natural convection, the convection in the radial direction should be insignificant [37]. Thus, $\lambda_{total}$ can be estimated according to Equation (1):

(1) $$\lambda_{total}=\lambda_{s}+\lambda_{g}+\lambda_{r}$$

The gas-phase thermal conductivity is expressed as follows:

(2) $$\lambda_{g}=\frac{\lambda_{g0}\Pi }{2\beta Kn+1}$$

(3) $$Κn=\frac{\Lambda_{g}}{D}$$

**Figure 5:**
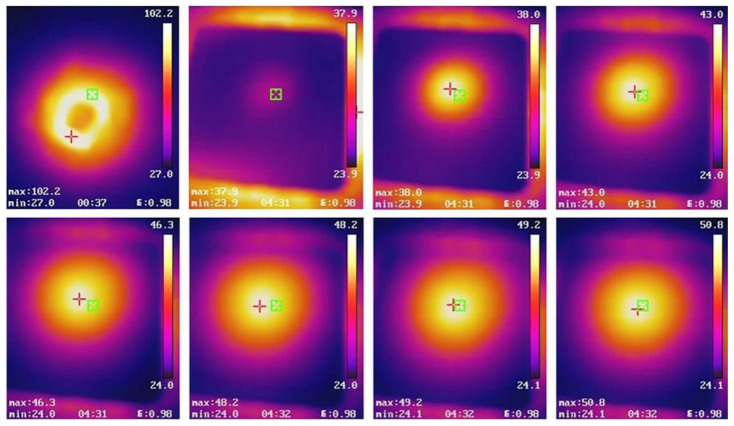
Infrared images of the DW/PI-60 aerogel at a point heat source of 102 °C.

In Equation (2), β(≈2 for air) is a coefficient dependent on the energy accommodation coefficient and the adiabatic coefficient of the gas in aerogels, and $K_{n}$ is the split of the mean free path ($\Lambda_{g}$ ≈ 70 nm) of air to the pore diameter (D) of the samples. Π denotes the porosity and $\lambda_{g0}$ is the gaseous conductivity in free space (0.025 W/(m·K)). According to Equations (2) and (3), the gaseous conductivity is strongly dependent on the pore size. Apart from that, solid conductivity largely relies on the density and the inherent property of the solid. In general, the resulting wood-based composite with a hierarchical pore structure can effectively reduce gas conduction owing to the presence of nanopores, which are less than the free path of air (~70 nm) [29,38]. The radiation $\lambda_{r}$ is minor because cell walls and fiber reduce the absorption and scattering [25]. On the other hand, the PI aerogel and cellulose nanofiber can serve as heat barriers, and therefore the solid conduction of pore walls can be reduced because of the increased interfacial thermal resistance [39,40,41]. Furthermore, the lower density contributes to inhibiting solid conduction. As the concentration of the PI networks increases, the bulk density of the composites increases from 0.053 (DW) to 0.148 g/cm^3^, resulting in a slight increase in the bulk thermal conductivity from 0.032 to 0.037 W/(m·K), which is still much lower than that of NW (0.066 W/(m·K)). Moreover, the thermal conductivity of DW/PI-60 was compared to those of various existing wood-based materials (containing cellulose as their main component), such as NFC/MHNPs [42], cellulose-derived aerogel [43], pineapple leaf/cotton-based aerogel [44], cotton/natural fiber-based aerogel [45], CNF/Al (OH)_3_/Na_2_SiO_3_ [46], etc. (Table S3 of Supplementary Materials). By comparison, the DW/PI-60 exhibited a lower thermal conductivity (0.034 W/(m·K)) than other cellulose-based composites in the references. The excellent thermal insulation properties of the DW/PI composite are further investigated using infrared images recorded at a fixed period. The temperatures of NW and DW/PI-60 were recorded every 10 s as shown in Figure 5 and Figure S4 of Supplementary Materials. The temperature of the point heat source was 102 °C. The sample is placed directly above the point heat source. The temperature on the upper surface of the sample was recorded every 10 s. At the heating time of 1 min, the temperature of NW is 57.9 °C, which is higher than that of wood/PI composite (50.8 °C), indicating good thermal insulation at high temperature for the wood composite. Based on these results, the wood-based composite is promising for energy management and improving energy efficiency.

### 2.4. Hydrophobicity

**Figure 6:**
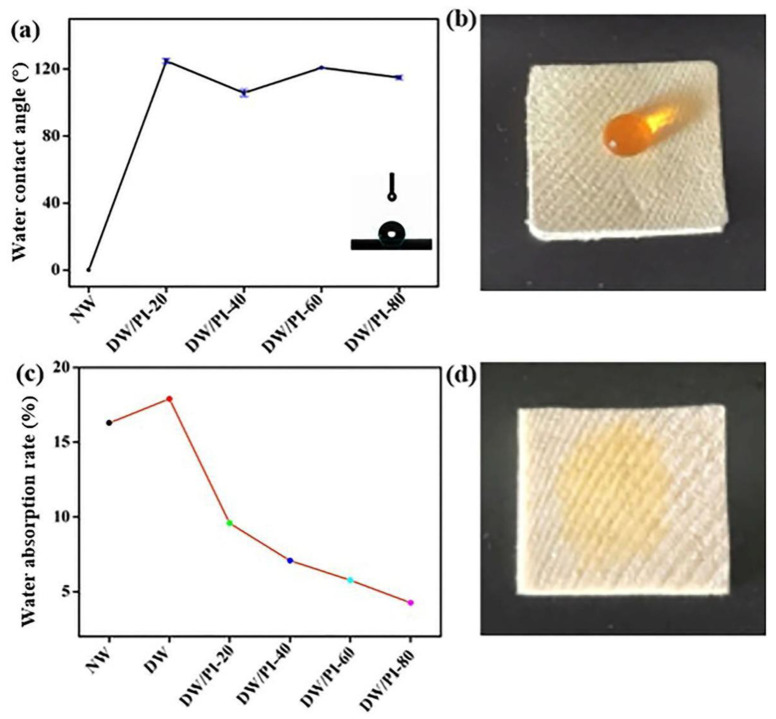
(**a**) WCAs of the samples; (**b**) water dropped onto the surface of the DW/PI-60 sample; (**c**) water absorption rate curve; (**d**) water dropped onto the surface of the NW sample. The water is colored orange with methyl orange before the experiments.

Hydrophobicity is an essential property of thermal insulation materials [47]. The water contact angles (WCAs) were measured to investigate the hydrophobicity of the NW and DW/PI composite. As shown in Figure 6d, the NW sample with cellulose nanofiber exposed on the surface exhibited high hydrophilicity. The addition of PI increased the hydrophobicity of the wood, as indicated by the high contact angle of 125° (Figure 6a). Videos S1–S3 of Supplementary Materials displays the hydrophobic properties of the samples. These results indicate that the fluorine-containing monomer of PI reduces the surface energy of materials as well as microstructure increases in surface roughness, therefore allowing the water droplets to slide away when the sample is slightly tilted. Based on the Wenzel’s model [48,49,50], the chemical composition and geometrical microstructure both influence the wettability, as the thermodynamic equilibrium (Equation (4)) shows:

(4) $$\cos\theta^{\omega }=\gamma \cos\theta$$

In Equation (4), γ stands for the roughness factor of the surface, θ is the Young’s angle, and $\theta^{\omega }$ denotes the apparent contact angle. The fluorine functional groups chosen as precursor effectively decrease the surface energy, as well as the surface roughness of the wood, thereby improving the hydrophobicity of the composite. To further verify the dimensional stability of the sample in an aqueous environment, we treated several samples in saturated water vapor at 25 °C for 24 h to obtain a water absorption rate curve (Figure 6c). It is noteworthy that both NW and DW show strong hydrophilicity, and the water absorption rates are 16.29% and 17.9%, respectively, which can be attributed to the increased pore sizes after removal of lignin and hemicellulose, thereby favoring the capillary effect. The recombination of PI effectively reduced the water absorption capacity of the sample. With the increase in PI introduction, the water absorption rate of the composite material decreases simultaneously. The water absorption rate of DW/PI-80 is only 4%, far lower than that of NW. Therefore, the obtained wood composites can be used in a high-humidity environment.

### 2.5. Thermal Stability and Fire Resistance

**Figure 7:**
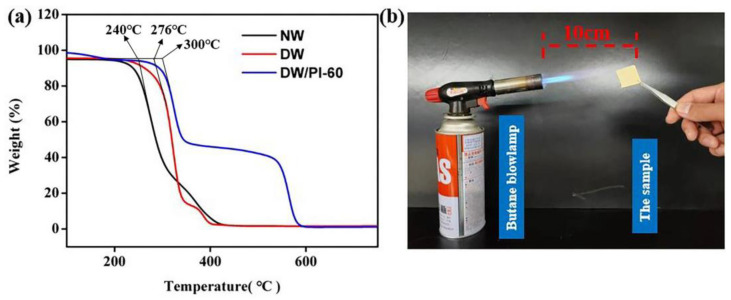
(**a**) TGA curves (measured in the air: the volume fraction of nitrogen is about 78%, oxygen is about 21%, carbon dioxide is about 0.04%, and the remaining is a rare gas), and (**b**) the combustion experiment.

The wood/PI composite exhibited excellent thermal stability owing to the impregnation of thermostable polyimide aerogel. The thermogravimetric analysis (TGA) curves of the samples (Figure 7a) revealed the thermal decomposition of the materials. For the NW sample, the pyrolysis process (220–310 °C) is mainly attributed to the degradation of lignin and hemicellulose. The second stage from 310 to 400 °C corresponded to the decomposition of cellulose. For the DW sample, the mass loss of wood was relatively low owing to the removal of hemicellulose and lignin. For the DW/PI-60 composite, the maximum decomposition rate appeared at 300 °C, which was much higher than that of NW (240 °C). The second stage of DW/PI-60 was between 310 and 360 °C, and the maximum thermal degradation rate appeared at 350 °C. The weight loss of the DW/PI-60 in these stages was ~55%, lower than that of NW and DW. The final stage (530–600 °C) was mainly the decomposition and mass loss of PI. These results show that the introduction of PI greatly increases the degradation temperature of the wood composite. In order to further compare the burning behavior of the DW/PI composite, NW, and common commercial insulators, the materials were exposed to an open butane blowlamp flame (~1300 °C). The combustion experiment was recorded (Figure 7b). The NW and DW/PI-60 were removed from the flame at the same time, and NW continued combusting until burnt to ashes, while the DW/PI-60 composite can extinguish automatically, indicating that the composite is fire retardant and can maintain its residual component intact after combustion (videos S4 and S5 of Supplementary Materials). However, commercial thermal-insulation materials, for example, PU foam and EPS, instantly burn violently in flames (videos S6 and S7 of Supplementary Materials), which poses safety issues. The exceptional fire retardance of the wood/PI composite can be attributed to two reasons: (1) In terms of chemical composition, the rigid main chain of PI obtained from fluorine-containing groups has high glass-transition temperature and heat resistance (>380 °C); and (2) structurally, the 3D networks of the PI aerogel inhibit the diffusion of thermal decomposition products and oxygen, thereby suppressing combustion.

## 3. Conclusions

We developed a fire-retardant, water-resistant wood composite aerogel via impregnation and using the ‘in situ gel’ method. In this process, lignin and hemicellulose were almost removed without destroying the aligned cellular scaffold, thereby allowing the fast impregnation of PAA and the ‘in situ gel’ process. Thus, the resulting wood composite had a hierarchical pore structure as well as excellent chemical compatibility. The hierarchical pore structure is composed of wood micron-scale lumen and PI nano-scale 3D networks, in which PI chains act as the “adhesive” for the replacement of lignin and hemicellulose. Benefiting from strong hydrogen bonds between the cellulose molecules and PI chains as well as the inherent comprehensive properties of PI components, the wood composite exbibits various attractive properties: (1) the wood composite exhibits excellent hydrophobicity (the highest water contact angle of 125°) and fire-retardant properties, and no considerable change in its mass was observed after saturation with water vapor at 25 °C for 24 h; (2) the composite had low thermal conductivity (DW/PI-60: ~0.034 W/(m·K)), which was almost half of that of NW(~0.066 W/(m·K)); (3) the composite improved mechanical properties (radial compressive strength of DW/PI-80:1.25 MPa, when the elastic strain of materials ε = 60%), the radial compressive strength of which increases by 5.7 times compared with that of DW; (4) the composite also exhibited a self-extinguishing property. When DW/PI-60 was removed from direct flame, the ignited fire of the sample extinguished automatically after 8 s, and the complete structure was retained. The developed wood composite as a kind of insulation material does not only possessing excellent thermal insulation properties, but also outstanding dimensional stability, flame retardation, and hydrophobicity. In the following study, the high-temperature insulation performance of composite materials will be investigated systematically to meet the insulation needs of industrial pipelines (such as steam pipelines). Therefore, it is promising for a wide range of application prospects.

## 4. Materials and Methods

### 4.1. Materials and Chemicals

Balsa wood was purchased from Guangzhou Sinokiko Balsa Co., ltd. Sodium hydroxide (NaOH, >95%), sodium sulfite (Na_2_SO_3_, >98%), sodium chlorite (NaClO_2_, 80%), ethylene glycol (≥99%), N-methyl-2-pyrrolidone (NMP, 98%), pyridine (>99%), 1,2,4,5-benzenetetracarboxylic anhydride (PMDA, 99%), and 2,2′-Bis(trifluoromethyl)benzidine (TFMB, 98%) were provided by Macklin. The dehydrating agent acetic anhydride (AR) was purchased from Sinopharm. The crosslinker 1,3,5-tris (4-amino phenoxy) benzene (TAB, ≥98%) was purchased from Aladdin. Deionized water (DI) was self-made in the laboratory. All reagents were used without further purification.

### 4.2. Preparation of Delignified Wood (DW)

Two pieces of natural balsa wood blocks (30 × 30 × 5 mm^3^) were immersed in 50 mL mixture solution of NaOH (2.5 M) and Na_2_SO_3_ (0.4 M) and kept for 12 h at 100 °C, followed by processing in an NaClO_2_ solution (pH = 4.7) for 3 h at 100 °C to completely remove the residual lignin. Then, the samples were immersed in deionized water to remove the chemicals. The obtained white wood samples were preserved in a freeze dryer and dried for 24 h to obtain the DW aerogel.

### 4.3. Preparation of DW/PI Composites

The as-prepared composite wood obtained by adjusting the PI composite ratio was defined as DW/PI-x. Here, x refers to the concentration of polyimide precursor solution (x = 20, 40, 60, and 80 mg/cm^3^). Furthermore, 1,2,4,5-Benzenetetracarboxylic anhydride (PMDA) and 2,2′-Bis(trifluoromethyl)benzidine (TFMB) were mixed with a molar ratio of 1.05:1 in an N-methyl-2-pyrrolidone (NMP) solvent, the concentration of the polymer in the solution (named solution A) was 10 wt%. After vigorous magnetic stirring for 2 h at room temperature, a certain amount of crosslinker 1,3,5-benzene tricarboxylic acid (BTC) was added to the solution, and the molar ratio between BTC and TFMB was controlled at 1:40. After 30 min of polymerization at room temperature, the solution was diluted to a certain concentration (2, 4, 6, and 8 wt%, respectively). Then, the DW samples were added into the solution and kept continuously immersed for 30 min. In the meantime, acetic anhydride and pyridine were dissolved in the NMP, which was subsequently added to the polymer solution, and stirred for 10 min, (the resulting solution—solution B). The samples impregnated with solutions of different concentrations were transferred into solution B, and remained for 24 h, followed by multi-step water-ethylene glycol solvent replacement of the samples to remove the chemicals. Then, the obtained samples were preserved in a freeze dryer and dried for 24 h to obtain the DW/PI composites.

### 4.4. Characterization

The morphology and structure of the wood samples were characterized by field-emission scanning electron microscopy (SEM, Thermo Scientific Apreo 2C, Thermo Scientific, Waltham, MA, USA). FT-IR spectra were recorded on a Thermo Scientific Nicolet iS10 (Thermo Scientific, USA) spectrometer. The crystalline structures were measured using an Ultima IV X-ray diffractometer (Rigaku Corporation, Tokyo, Japan) in the range of 5–40° (2θ). The mechanical compressibility of the samples was measured using a universal testing machine (WDW-D1000N, Jinan Xinguang Testing Machine Manufacturing Co., Ltd., Jinan, China). Thermal conductivity of the samples was evaluated using a C-Therm TCi thermal conductivity analyzer (Fredericton, NB, Canada) through a transient plate method. The thermal degradation behavior of the samples in air was investigated using thermogravimetry analysis (TGA5500, USA). Static contact angles of the samples were performed by means of the contact angle measuring system (OCA 50, Dataphysics, Stuttgart, Germany) at room temperature. Infrared imaging was performed using a thermal infrared camera (H10, HIKMICRO). Acid-insoluble lignin content in the samples was determined with a 72 wt% sulfuric acid solution according to the GB/T 20805-2006 standard; holocellulose and α-cellulose contents were measured in accordance with the GB/T 20806-2006 standards, respectively. A list of abbreviations of the main text is shown in Table A1 (Appendix A).

## Data Availability

The data presented in this study are available on request from the corresponding author.

## Supplementary Materials

The following supporting information can be downloaded at: https://www.mdpi.com/article/10.3390/gels9060467/s1, Video S1: Video of the process of hydrophobicity test of NW; Video S2: Video of the process of hydrophobicity test of DW; Video S3: Video of the process of hydrophobicity test of DW/PI-60; Video S4: Video of the combustion process of NW; Video S5: Video of the combustion process of DW/PI-60; Video S6: Video of the combustion process of polyurethane (PU) foam; Video S7: Video of the combustion process of polyethylene benzene foam (EPS); Figure S1: SEM images of NW and DW; Figure S2: Process of fabrication of the DW/PI aerogel; Figure S3: Infrared images of the NW sample and the experimental setup; Table S1: The properties of NW, DW, and the composites with different PI content; Table S2: Comparison of the mechanical strength of wood/PI-80 with conventional thermal insulation materials; Table S3: Comparison of the thermal conductivity of wood/PI-60 with various existing wood-based materials (containing cellulose as main component). The porosity of DW/PI-x composites was calculated according to Equation (S1) [26,35,36,42,43,44,45,46,51,52].

## Appendix A

A list of abbreviations with transcripts is shown in Table A1.

**Table A1:** List of abbreviation in the article.

Abbreviation	Full Name
PAA	polyamic acid
EPS	expanded polystyrene
PU	polyurethane
PI	polyimide
NW	natural wood
DW	delignified wood
3D	three-dimensional
DW/PI	delignified wood/polyimide
ε	strain
XRD	X-ray diffraction
FT-IR	Fourier transform-infrared spectrometer
SEM	scanning electron microscope
WCAs	water contact angles
$\lambda_{total}$	total thermal conductivity
$\lambda_{g}$	gas conduction
$\lambda_{s}$	solid conduction
$\lambda_{r}$	thermal radiation
$\lambda_{g0}$	gaseous conductivity in free space
β	a coefficient (≈2) for air in aerogels
${Κ}_{n}$	Knudsen number
$\Lambda_{g}$	mean free path (Λg ≈ 70 nm) of air
D	diameter
Π	porosity
TGA	thermogravimetric analysis
