# Supplementary material for: Robust, Fire-Retardant, and Water-Resistant Wood/Polyimide Composite Aerogels with a Hierarchical Pore Structure for Thermal Insulation
Source: Gels. 2023 Jun 6;9(6):467. doi: 10.3390/gels9060467 (PMC10298113; doi:10.3390/gels9060467)
Supplement: Supplementary file 1 [file gels-09-00467-s001.zip › gels-2397944-supplementary.pdf]

gels

MDPI

Supplementary Materials

Robust, Fire-Retardant, and Water-Resistant Wood/Polyimide Aerogel with a Hierarchical Pore Structure for Thermal Insulation

Lu Zhao †, Junyong Chen *,†, Defang Pan and Yan Hou

This supplementary information contains:
1. Supplementary movies
2. SEM images of NW and DW
3. Process of fabrication
4. Infrared images of the NW sample and the experimental setup
5. Physical properties of the samples
6. Comparison of the mechanical strength of wood/PI-80 with conventional thermal insulation materials
7. Comparison of the thermal conductivity of wood/PI-60 with various existing wood-based materials (containing cellulose as main component)

1. Supplementary Movies

Movie S1

Movie of the process of hydrophobicity test of NW.

Movie S2

Movie of the process of hydrophobicity test of DW.

Movie S3

Movie of the process of hydrophobicity test of DW/PI-60.

Movie S4

Movie of the combustion process of NW.

Movie S5

Movie of the combustion process of DW/PI-60.

Movie S6

Movie of the combustion process of polyurethane (PU) foam.

Movie S7

Movie of the combustion process of polyethylene benzene (EPS) foam.

2. SEM images of NW and DW

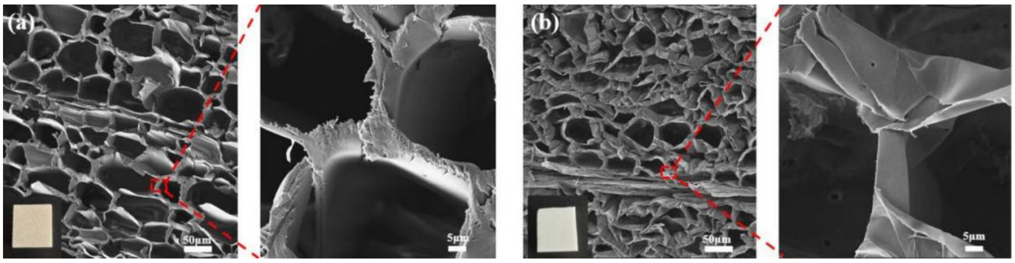

Figure S1. SEM image of (a) NW and (b) DW.

3. Process of fabrication

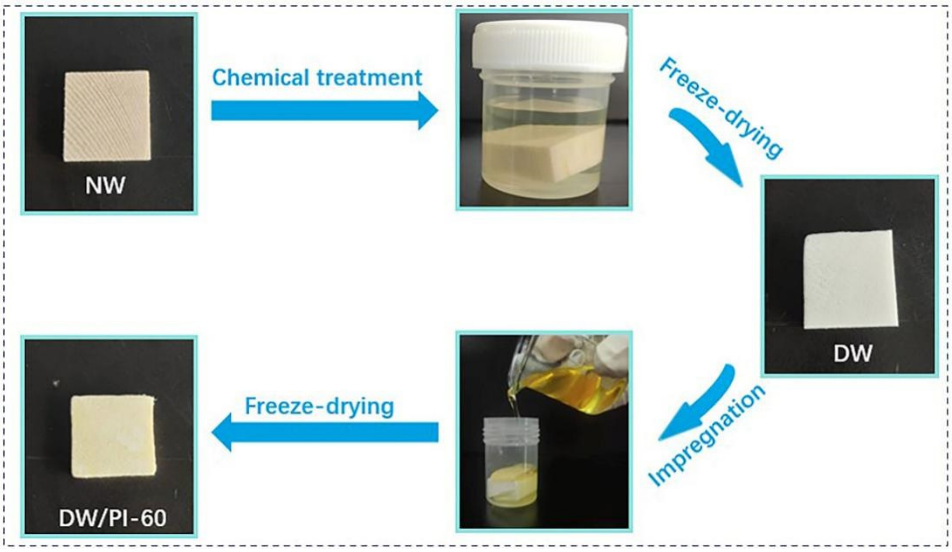

Figure S2. Process of fabrication of the DW/PI aerogel.

4. Infrared images of the NW sample and the experimental setup

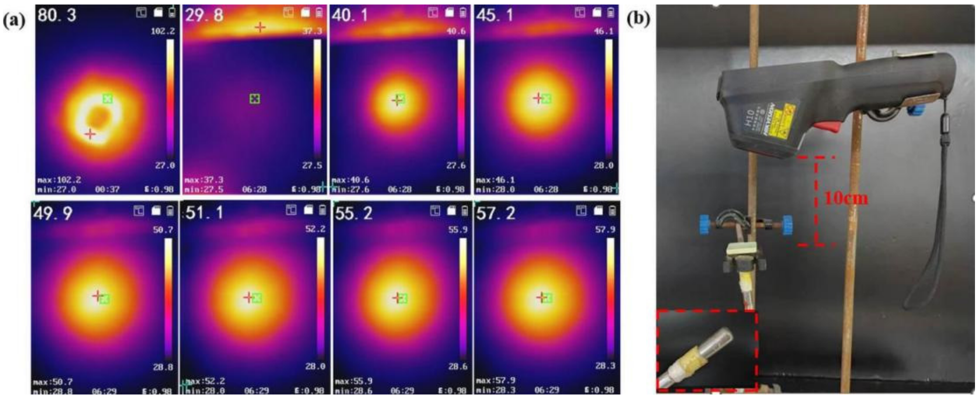

Figure S3. (a) Infrared images of the NW at a point heat source of 102.2 ℃ (b) the experimental setup; inset is the point heat source.

5. Physical properties of the samples

Table S1. The properties of NW, DW, and the composites with different PI content.

Samples	Density (g· cm^{-3})	Thermal conductivity (W /m·K)	Porosity (%)	Compressive strength (MPa)
NW	0.094	0.066	93.7	1.1
DW	0.053	0.032	96.5	0.218
DW/PI-20	0.074	0.038	97.0	0.38
DW/PI-40	0.095	0.035	93.5	0.618
DW/PI-60	0.119	0.034	91.7	0.9
DW/PI-80	0.148	0.037	89.7	1.25

Measuring the weight and volume of each individual sample can calculate the apparent volumetric mass density of the sample. And according to eq (S1), where ρ, ρ_s, and ρ_c refer to the bulk density of DW/PI-x composite and the skeleton densities of pure PI aerogels and wood. ωs and ωc represent the mass fraction of PI and wood in the DW/PI-x aerogels, respectively. Herein，ρ_s , and ρ_c were designed as 1.4 g·cm^{-3} and 1.5 g·cm^{-3} [51,52].

$$porosity(\%) = \left(1 - \frac{\rho}{\omega_s\rho_s + \omega_c\rho_c}\right) \times 100\% \tag{S1}$$

6. Table S2. Comparison of the mechanical strength of wood/PI-80 with conventional thermal insulation materials.

Samples	Density (g· cm^{-3})	Compressive strength (MPa)	Reference
wood-waste foam	0.036	0.0528	[35]
stone wool	0.034	0.06	[36]
EPS foam	0.016	0.045	[26]
DW/PI-80	0.148	1.25	This work

As shown in Table S2, when the strain is 60% in the radial direction, the wood/PI-80 in this work has an advantage of high compressive strain reaches up to 1.25 Mpa, which is much higher than other reported conventional thermal insulation materials, such as wood-waste foam, stone wool, and EPS foam.

7. Table S3. Comparison of the thermal conductivity of wood/PI-60 with various existing wood-based materials (containing cellulose as main component).

Samples	Density (g· cm^{-3})	Thermal conductivity (W m^{-1}K^{-1})	Reference
NFC/MHNPs	—	0.056-0.081	[42]
Cellulose derived aerogel	0.05-0.109	0.040-0.0532	[43]
Pineapple leaf/cotton-based aerogel	0.019-0.046	0.039-0.043	[44]
Cotton/natural fiber-based aerogel	0.028-0.105	0.036-0.0473	[45]
CNF/$Al(OH)_3$/Na_2SiO_3	—	0.045	[46]

DW/PI-80	0.148	0.037	This work

Furthermore, we compared thermal conductivity of wood/PI-60 with various existing wood-based mterials(containing cellulose as main component) such as, NFC/MHNPs, Cellulose derived aerogel, Pineapple leaf/cotton based aerogel , Cotton/natural fiber based aerogel, CNF/Al(OH)$_3$/Na$_2$SiO$_3$ etc.(Table S3). The DW/PI-60 exhibited a lower thermal conductivity (0.034 W m^{-1} K^{-1}) than wood derived aerogel and cellulose derived aerogel.
